# Exposure to alcohol advertising and alcohol consumption among children and early teenagers in South Africa

**DOI:** 10.1186/s13104-023-06364-5

**Published:** 2023-07-12

**Authors:** Godswill N. Osuafor, Chinwe E. Okoli, Gladys Chibuzor

**Affiliations:** 1 Department of Population Studies and Demography, North West University, Mafikeng Campus, Mafikeng, South Africa; 2 Federal Medical Centre, Umuahia, Nigeria; 3 Centre for advocacy on drugs and substance abuse, Port Harcourt, Nigeria

**Keywords:** Alcohol, Advertising, Children, Early teenagers, South Africa

## Abstract

**Background:**

There is a paucity of information on alcohol adverts on alcohol consumption among early teenagers. The study aimed to examine the association between exposure to alcohol adverts and alcohol consumption.

**Methods:**

A sample of 3833 early teenagers aged 12–14 years were extracted from the South African National HIV Prevalence, HIV Incidence, Behaviour and Communication Survey (SABSSM) 2017. Participants answered questions related to their exposure to alcohol adverts across television, Poster/billboard, events, and social media. Alcohol consumption was assessed on ever consumed alcohol and in the previous 12 months. Information on demographic characteristics was collected. Descriptive and inferential statistics were used to process the data.

**Result:**

Exposure to alcohol adverts increases from 5.2% for Events to 77.0% on television. All alcohol media of adverts were associated with alcohol consumption by age. The results mirror studies that demonstrated that exposure to alcohol advertisements was associated with alcohol consumption.

**Conclusion:**

The association between alcohol consumption, exposure to alcohol adverts, and demographic characteristics need the urgent attention of policymakers and interventionists. The focus of action should be on protecting these early teenagers from exposure to media of adverts and risk background factors.

## Introduction

In low and middle-income countries, the prevalence of alcohol uses at least once in the past 30 days was 8.4% among early teenagers aged 12–13 years, [1]. The Youth Risk Behaviour Survey (YRBS) 2013 showed that 16% and 8.7% of male and female teenagers respectively have started alcohol consumption before 13 years of age. In a longitudinal study to delay alcohol use and sexual initiation among learners, alcohol consumption increased from 34.6 to 39.9% between the two points [2]. The study further revealed that the proportion of learners reporting alcohol consumption was 45.6% and 26.4% among males and females respectively. Studies elsewhere have shown that testing and sipping of alcohol began between the age of 10 to 14 or even younger [3, 4]. There is evidence that the strongest association between underage drinking and alcohol availability was found among children aged 12–14 years [5, 6].

Alcohol remains one of the main three leading health factors for global disease burden [7], with underage alcohol consumption presenting grave health problems. Three million death was attributed to alcohol use annually in 2016 [8]. Studies have associated alcohol drinking with mental health disorders, suicidal behaviour [9, 10], Chronic liver disease [10, 11] and violence [12–14]. A national longitudinal study of adolescents from grade 7 to grade 12, showed that early initiation of alcohol use translated to a higher level of alcohol use in adulthood [15]. Other studies have reported alterations in the maturation and functioning of the adolescent brain because of early exposure to alcohol use [16, 17].

Advertising alcohol has been recognised as an instigator of alcohol misuse and health risk to drinkers. Several studies have associated exposure to alcohol advertisements with alcohol consumption among adolescents [18–22]. In South Africa, a qualitative study conducted among 40 high school learners showed that alcohol consumption was motivated by alcohol advertisements in Eastern Cape [23]. In another study of alcohol availability and marketing among rural and urban youth in South Africa, [24] reported that consumption of alcohol and binge drinking were induced by alcohol advertising and marketing. A systematic review of alcohol adverts and prospects for alcohol consumption in developing countries showed that alcohol advertising predicted initiating alcohol drinking among children and youths [25]. Similar evidence of alcohol adverts leading to alcohol consumption among children and adolescents has been documented in the US [26], Thailand [27, 28] and Australia [22]. Self-reported advertisement exposure on four types of media (TV, radio, magazine and billboards) was associated with increased drinking over time among 1877 youths in multilevel analytical studies in which other covariates were controlled [29].

However, Gunter et al.  [30] did not find any association between exposure to any type of alcohol advertising (cinema, magazine, TV) and general alcohol consumption. In a randomized control trial study, exposure to alcohol advertisements in grade six did not predict drinking behaviour amongst grade six drinkers or grade eight non-drinkers [31]. In addition, Jones demonstrated that other factors play a more decisive role in alcohol initiation among young people than alcohol advertisements in Australia [22]. Furthermore, in many parts of the world, the locally-made alcohol that people consume was never advertised [32]. These suggest that the association between alcohol advertisement and consumption may be mediated through other factors.

There is evidence that a spectrum of other factors in the environment of young people promotes alcohol consumption. Risk factors studies have documented parental drinking behaviour [28, 33–35] the community where the adolescents were living [28] gender [28] and direct peer group influences [28, 30, 36, 37] were associated with adolescents’ alcohol use. Other studies have demonstrated an association between alcohol consumption and ethnicity [19, 32]. Taking together the association between alcohol advertisement and alcohol consumption appeal to a multifaceted investigation among children and early teenagers.

Exposure to media alcohol advertisements has been documented to influence alcohol consumption among young people in South Africa. Qualitative empirical studies [23, 24] exist on the interaction between alcohol advertising and drinking among young people in a few provinces of South Africa. However, qualitative studies were limited in accounting for the association between alcohol adverts and alcohol use among children and early teenagers in South Africa. Morojele et al. [38] examine the role of demographic factors, exposure to alcohol marketing and linking of alcohol advertisements in predicting the use of alcohol in the past 6 months among 408 males and 461 females aged 16 and 17 years old in Tshwane Municipality. They found a positive association between the mode of alcohol exposure with alcohol use in the past 6 months. However, the study population did not account for early teenagers aged 12 to 14 years. Furthermore, the geographical coverage and 6 months assessment of the study was very limited and a short period to account for a nationally representative population of alcohol exposure and underage drinking. Hence, these studies cannot stimulate policy on alcohol adverts and underage drinking in South Africa. Thus, there exists a paucity of information between alcohol adverts and alcohol consumption among children and early teenagers. South Africa has regulations that forbid the selling of alcohol to people below the age of 18. Furthermore, the Bill to ban alcohol advertisements to improve the health of children and teenagers below the age of 18 has not been passed into law because of various competing interests. Some are concerned that banning alcohol adverts would affect economic activities and subsequently lead to job losses [39]. On 26th June 2022, twenty-one teenagers comprising 12 girls and 9 boys aged 13–17 were found dead at the Enyobeni Tavern in Scenery Park in East London on account of alcohol-related. Sacrificing the health and lives of early teenagers on the altar of economic viability and job sustenance has been a difficult stand for both researchers and policymakers concerning alcohol advertisement. There is evidence that alcohol advertisement in media affects alcohol consumption. However, this evidence is sketchy for children and early teenagers. Furthermore, it is not clear whether all forms of media advertisement for alcohol mediate alcohol consumption among these early teenagers. In addition, other background factors have been shown to be more predisposing to underage alcohol consumption. The study is a secondary data analysis extracted from the South African National HIV Prevalence, HIV Incidence, Behaviour and Communication Survey (SABSSM) 2017. The survey is conducted every three years among individuals of all ages. This enables exploration of shifts over time on demographic and other variables, as well as allowing for investigation of new research areas such as exposure to underage alcohol exposure and consumption. Hence the dataset has robust information on the study population and dynamics in alcohol use. The study aimed to examine the association between exposure to alcohol advertising media and underage alcohol consumption while controlling for the background characteristics in South Africa.

## Methods

The South African National HIV Prevalence, HIV Incidence, Behaviour and Communication Survey (SABSSM) 2017 was a cross-sectional, population-based household survey conducted using a multi-stage stratified random cluster sampling approach [40]. SABSSM 2017 was the fifth wave in a series of national cross-sectional surveys that collected information from all population segments using households and hostels as visiting points from December 2016 to February 2018. Educational institutions, old-age homes, hospitals, and uniformed-service barracks were excluded from the sample.

The 2017 survey used small area layers (SALs) as the primary sampling unit (PSU) instead of the enumeration areas (EAs) to bypass the confidentiality issues associated with EAs having a few VPs. A SAL consists of one or more EAs and is better defined geographically than the EA [41]. Four questionnaires used in SABSSM 2017 comprised a Household questionnaire (also known as a visiting-point questionnaire), a questionnaire for parents and guardians of children aged 0–11 years, a questionnaire for children aged 12–14 years and a questionnaire for people aged 15 years and older.

However, the present study used a dataset from the questionnaire for children aged 12–14 years. The participants in the present study were 3833 early teenagers aged 12 to 14 years old. Nearly half of the participants (47.8%) were males. Information collected on race was categorised into Africa, coloured, Indian, and white. Over two-thirds (75.4%) were African. Information on the nine South African provinces was recategorized into rural and urban provinces which showed that over a quarter (29.4%) were residing in urban designated provinces.

Participants were asked about where they have seen alcohol adverts: Whether on television, poster/billboard, social media (internet, Facebook, Whatsup, Wechat) and Events. These were coded as (Yes, No). Posters and billboards were combined, and the components of social media were also merged. They were further asked whether they have ever consumed alcohol (Yes, No). In addition, recent alcohol consumption was accessed by asking about alcohol consumption in the past 12 months (Yes, No).

### Statistical analysis

The background characteristics, alcohol exposure and alcohol consumption were examined with the age of the respondents. The relationship between alcohol advertising and ever alcohol consumption and recent alcohol consumption was examined by logistic regressions. Unadjusted model 1 for each type of alcohol advertising media and alcohol consumption was examined. In model 2 all the media of alcohol advertisements and alcohol were examined. In model 3, exposure to all alcohol advertising media and ever alcohol consumption and recent alcohol consumption were examined while controlling for age, gender, race, and province. The analyses at the multivariate level were carried out on the weighted sample to account for the population of children and early teenagers in South Africa. The results were presented as unadjusted and adjusted odds ratios with p values < 0.05 indicating statistical significance. All the analyses were carried out using IBM SPSS version 27.

## Results

Table 1 presents the media of exposure to alcohol advertisement and alcohol consumption among the early teenager sample. Seeing alcohol advertisements on television was reported by nearly one-third (23.0%). Over three quarters (71.6%) and nearly all participants (94.8%) indicated that they have seen alcohol advertisements on posters/billboards and events. About 9 in 10 have seen alcohol advertainments on social media. Over three quarters (74.5%) indicated that they have consumed alcoholic drinks while in the last 12 months about half (51.9%) have consumed alcohol. About 7 in 10 have engaged in sexual intercourse.

**Table 1:** The percentage distribution of early teenagers’ exposure to alcohol advertisement and consumption by age

	Age			
	12 n = 1452	13 n = 1263	14 n = 1118	Total n = 3833
Seen alcohol advertisements on television				
No	23.9	21.9	23.2	23.0
Yes	76.1	78.1	76.8	77.0
Seen alcohol advertisement on a poster/billboard				
No	73.8	71.6	68.9	71.6
Yes	26.2	28.4	31.1	28.4
Seen alcohol advertisements at events				
No	95.9	93.5	94.9	94.8
Yes	4.1	6.5	5.1	5.2
Seen alcohol advertisements on social media				
No	94.4	92.6	89.0	92.2
Yes	5.6	7.4	11.0	7.8
Ever consumed alcohol				
No	75.4	71.9	76.4	74.5
Yes	24.6	28.1	23.6	25.5
Consumed alcohol in the past 12 months				
No	64.1	47.8	47.9	51.9
Yes	35.9	52.2	52.1	48.1

### Association between media of alcohol adverts and ever-consumed alcohol

Table 2 shows seeing alcohol advertisements in television and posters/billboard was associated with alcohol consumption which intensity from [OR = 1.64 (1.63–1.66) to AOR = 3.28 (3.22–3.34)] for television and from [OR = 1.38 (1.37–1.40) to AOR = 1.52 (1.52–1.54)] for posters/billboard. However, reduced odds of consumption of alcohol 35% and 30% were reported among those who indicated seeing alcohol advertisements during events and on social media, which increased to 42% and 37% respectively after adjusting for background characteristics. Those aged 12 and 13 years old were more likely to report alcohol consumption compared to those aged 14 years. Furthermore, males showed 35% reduced odds of reporting alcohol consumption compared to their female counterparts. Compared to the African, Coloured, Indian, and White were less likely to report alcohol consumption. Teenagers residing in rural provinces were more likely to report alcohol consumption than their urban counterparts.

**Table 2:** The association between alcohol advertisement and ever alcohol consumption

	Model 1		Model 2		Model 3	
	OR	95% CI	AOR	95% CI	AOR	95% CI
Advert on television						
No (ref)	1.00	1.63–1.66	1.00	1.81–1.86	1.00	3.22–3.34
Yes	1.64***		1.83***		3.28***	
Advert on a poster/billboard						
No (ref)	1.00	1.37–1.40	1.00	1.43–1.46	1.00	1.52–1.54
Yes	1.38***		1.45***		1.52***	
Advert on alcohol during events						
No (ref)	1.00	0.63–0.66	1.00	0.55–0.57	1.00	0.57–0.59
Yes	0.65***		0.56***		0.58***	
Seen alcohol advertisements on social media						
No (ref)	1.00	0.69–0.71	1.00	0.60–0.62	1.00	0.61–0.64
Yes	0.70***		0.61***		0.63***	
Age						
12					1.06***	1.04–1.07 1.29–1.33
13					1.31***	
14 (ref)					1.00	
Sex						
Female (ref)					1.00	0.64–0.66
Male					0.65***	
Race						
African (ref)					1.00	0.49–0.51 0.68–0.73 0.29–0.31
Coloured					0.05***	
Indian					0.70***	
White					0.29***	
Province						
Urban (ref)					1.00	1.23–1.27
Rural					1.25***	

*CI* confidence interval,* ref * Reference category, *OR* Odds ratio, *AOR A*djusted odds ratio

Significant at ***p < 0.001

### Alcohol advertainments and alcohol consumption in the last 12 months

Table 3 shows alcohol consumption in the last 12 months and the association with exposure to alcohol advertisements. Exposure to alcohol advertisements on television and posters/billboards was consistently associated with alcohol consumption in the last 12 months. Conversely, exposure to alcohol adverts during events and on social media showed reduced odds of reporting alcohol consumption. In the last 12 months, teenagers who were below the age of 14 years and males were less likely to report alcohol consumption. Compared to the African teenagers, the coloured and Whites were more likely to report alcohol consumption in the last 12 months.

**Table 3:** The association between alcohol advertisement and alcohol consumption in the last 12 months

	Model 1		Model 2		Model 3	
	OR	95% CI	AOR	95% CI	AOR	95% CI
Advert on Television						
No (ref)	1.00		1.00		1.00	1.00–1.09
Yes	2.17***	2.11–2.23	1.66***	1.61–1.72	1.05*	
Advert on a poster/billboard						
No (ref)	1.00		1.00		1.00	5.32–5.64
Yes	4.35***	4.25–4.45	5.33***	5.19–5.46	5.48***	
Advert on alcohol during events						
No (ref)	1.00		1.00		1.00	0.28–0.30
Yes	0.62***	0.60–0.64	0.38***	0.37–0.40	0.29***	
Seen alcohol advertisements on social media						
No (ref)	1.00		1.00		1.00	0.26–0.28
Yes	0.70***	0.68–0.72	0.53***	0.52–0.55	0.27***	
Age						
12					0.10***	0.09–0.11 0.52–0.55
13					0.53***	
14 (ref)					1.00	
Sex						
Female (ref)					1.00	0.71–0.75
Male					0.73***	
Race						
African (ref)					1.00	
Coloured					1.69***	1.64–1.74
Indian					–	
White					11.69***	11.95–12.48
Province						
Urban (ref)					1.00	1.61–1.72
Rural					1.66***	

*CI* confidence interval,* ref  *Reference category, *OR* Odds ratio, *AOR A*djusted odds ratio

Significant at ***p < 0.001

*p < 0.05

### Exposure to alcohol adverts and alcohol consumption according to age

Table 4 shows that after adjusting for the demographic characteristics, there was an association between alcohol consumption and exposure to alcohol advertising media. For all ages, alcohol adverts on television were associated with alcohol consumption [AOR = 3.46: (3.33–3.58)] for 12 years; [AOR = 1.50: (1.46–1.55)] for 13 years and [AOR = 7.61: (7.33–7.89)] for the 14 years old. A positive association was reported among those who reported exposure to alcohol adverts through posters/billboards. Alcohol advertising during events was associated with a reduced likelihood of alcohol consumption by age [AOR = 0.18: (0.17–0.19) for 12 years; [AOR = 0.71: (0.69–0.72)] for 13 years and [AOR = 0.69: (0.67–0.71)] for the 14 years old. Reduced odds 79%; 35% and 48% for consuming alcohol were reported by 12, 13 and 14 years old respectively for those who were exposed to alcohol adverts on social media. Males were less likely than females to report alcohol consumption across the ages. By racial divides, all were less likely to report alcohol consumption compared to the Africans among those aged 12 and 14 years. For those aged 13 years, apart from the Whites, other races were less likely than Africans to report alcohol use. Across all ages teenagers in the rural provinces were more likely than their urban counterparts to report alcohol consumption.

**Table 4:** The Association between advertising and alcohol consumption by age

	12 years				13 years				14 years			
	OR	95% CI	AOR	95% CI	OR	95% CI	AOR	95CI	OR	95CI	AOR	95CI
Advert on Television												
No	1.00	1.36–1.43	1.00	3.33–3.58	1.00	1.62–1.70	1.00	1.46–1.55	1.00	1.84–1.93	1.00	7.33–7.89
Yes	1.39***		3.46***		1.66***		1.50***		1.89***		7.61***	
Advert on a poster/billboard												
No	1.00	0.78–0.81	1.00		1.00	1.47–1.53	1.00	1.64–1.72	1.00	2.52–2.62	1.00	2.11–2.20
Yes	0.79***		1.19***	1.17–1.21	1.50***		1.68***		2.57***		2.16***	
Advert on alcohol during events												
No	1.00		1.00		1.00		1.00		1.00		1.00	
Yes	0.22***	0.20-0.23	0.18***	0.17–0.19	0.61***	0.59–0.64	0.71***	0.69–0.72	0.86***	0.84–0.89	0.69***	0.67–0.71
Seen alcohol advertisements on social media												
No	1.00	0.39–0.43	1.00	0.19–0.22	1.00		1.00	0.63–0.67	1.00	0.31–0.34	1.00	0.49–0.56
Yes	0.41***		0.21***		1.19***	1.16–1.22	0.65***		0.32***		0.52***	
Sex												
Female (R			1.00	0.61–0.63			1.00				1.00	0.68–0.71
Male			0.62***				0.68***	0.67–0.69			0.69***	
Race												
African (R			1.00	0.55–0.58 0.94–1.03 0.06–0.07			1.00	0.69–0.72 0.32–0.37 2.34–2.54			1.00	0.32–0.34 0.96–1.09 0.05–0.06
Coloured			0.57***				0.71***				0.33***	
Indian			0.99				0.34***				1.02	
White			0.06***				2.44***				0.05***	
Province												
Urban (R			1.00	1.19–1.24			1.00	1.03–1.08			1.00	1.75–1.86
Rural			1.21***				1.00				1.81***	

*CI* confidence interval,* ref  *Reference category, *OR* Odds ratio, *AOR A*djusted odds ratio

Significant at ***p < 0.001

## Discussion

Early teenagers consume alcohol despite regulations that forbid them from consuming alcohol in South Africa. These early teenagers have reported that they have been exposed to alcohol advertisements on television, on posters/billboards, on social media and during events. The present study has shown the patterns of influence different media of exposure to alcohol advertisements has on underage drinking in South Africa. The nature of association differed by the media of alcohol advertisements. The result of the study showed that alcohol advertisements on television and posters/billboards were associated with ever-consumed alcohol and recent alcohol consumption. This finding is consistent with reports linking TV and billboard alcohol advertisements to adolescents’ alcohol consumption in Zambia [42], Chile [43] and developing countries [25]. However, disagreed with studies in Australia [22] which did not find an association between alcohol adverts on posters/billboards and alcohol consumption in the last 12 months among teenagers aged 12–15 years. Furthermore, contradicts the findings that exposure to television advertisements was associated with reduced odds of alcohol consumption in the last 12 months in Australia [22].

Our results further revealed that exposure to alcohol advertisements on social media and during an event showed lower tendencies of ever-consumed alcohol and recent alcohol consumption. The possible explanation for this observation could be attributed to the small proportion of early teenagers that have been exposed to alcohol advertisements during events and on social media. Previous studies reported only 2.4% had never been exposed to alcohol-related content, on media [44] compared to 92.2% observed in the present study. Secondly, given the ages of these children, they may lack despotic opportunities to attend events and limited access to social media which impacts negatively their exposure to alcohol advertisements and subsequent prevention of underage drinking. Our results concerning social media contrast with the assumption that anything that enhances exposure to alcohol marketing may also lead to alcohol use [19]. Other studies have reported that social media exposure to adolescent alcohol use was mediated by normative beliefs or perceived norms [20, 45]. The patterns of the effects of alcohol advertisement exposures during events or through social media did not differ by age despite adjusting for other factors. These suggest that other factors play stronger roles in underage alcohol consumption than exposure to alcohol advertisements during events and social media. These findings are pertinent for an integrated strategy for the prevention of underage alcohol consumption.

The result of our findings is in line with a previous report in Thailand [28] that reported reduced odds of alcohol consumption among males. This is surprising given that males are always associated with alcohol drinking. This suggests that in the early teenage, there is an inverse association between alcohol consumption and the male gender. Our finding is further in consonance with the report that current drinking in the past year was associated with teenagers aged 14 years [28] compared to those aged 12 and 13 years.

Overall rural Provinces influenced early teenage alcohol consumption, a finding that concurs with a recent report in Spain [46] and Germany [47]. It is anticipated that the teenagers from the rural provinces would show more potency of alcohol consumption due to cultural rituals and the availability of locally made alcohol for traditional ceremonies. Again, unlicensed liquor outlets proliferating as apartheid legacy were common in rural provinces. Enacting a comprehensive national alcohol regulatory policy has remained at an impasse in South Africa. Hence each province is autonomous in regulating alcohol operations. The mysterious death of 21 teenagers in Enyobeni Tavern in East London, South Africa may suggest that the province is losing control over underage alcohol regulation. We attributed rural teenagers’ alcohol consumption to industry sponsorship of alcohol marketing which is promoted by free alcohol and permeating into the rural areas of South Africa. These findings suggest that stricter regulation of alcohol exposure in rural provinces is needed in South Africa.

## Conclusion

Our study on media exposure to alcohol adverts among children and early teenagers is novel various media examined were based on the national representative population of South Africa. To the best of our knowledge, no similar study has been conducted in South Africa. The findings of the study showed patterns of influence on alcohol consumption with the medium of exposure. The present study revealed that advertising alcohol through television and posters/billboards needs to be revisited for additional restrictions. On the other hand, exposure through social media and during events appeals for continuous monitoring as they currently do not pose threat to underage drinking. Further actions to deter children and early adolescents from alcohol consumption should incorporate background characteristics which are risk factors into a real-time prevention programme.

## Limitations

The current study provided information on the impact of different types of alcohol advertisements and underage drinking. However, interpretation should be done in light of some limitations. Given that the study population were children who are entirely under parental guidance, we did not have variables to measure parental factors in the study. We do not know to what extent parental guidance has played a role in the whole gamut of alcohol use. However, a systematic review of studies documented several parental factors such as parental modelling, limiting the availability of alcohol to the child, disapproval of adolescent drinking, general discipline, parental monitoring, parent-child relationship quality, parental support, and general communication [45, 46] were deterrents to underage drinking. A recent study has also reported that parental support and monitoring influence adolescent alcohol use [47]. Variables to examine peer influences were also missing from the survey data. Furthermore, knowledge about alcohol use was not assessed, hence we were unable to account for the knowledge gap in the study. However, future research will take note of parental factors, peer influences and knowledge.The study was a cross-sectional design which limited our ability to establish causal inference. The findings of the study remain extant in the current environment because children and early teenagers are constantly exposed to alcohol advertisements in South Africa.

## Data Availability

Data are available on. The datasets generated and analysed during the current study are available. The data is available through the HSRC Research Data Service (http://datacuration.hsrc.ac.za/).
